# Disease of the Lungs

**Published:** 1901-04-06

**Authors:** 


### Disease of the Lungs.

(*Continued from page* 454.)

**Prognosis.**—Taylor[7] adopts the Italian nomenclature of polyorromenitis for those cases which show multiple inflammation of serous membranes. He quotes the Italians' conclusions with regard to ætiology, course and treatment of these cases. It is more frequent in males, usually between 16 and 30 years. The invasion usually occurs in the order of frequency:—

(1) The peritoneum first, then the pleuræ, beginning generally with the right.

(2) The pleuræ, then the peritoneum.

(3) The pleura on one side, then that on the other.

(4) Pericarditis usually follows a pleurisy, and especially a left-sided pleurisy.

(5) The inflammation may first involve one pleura, then the peritoneum, then the other pleura.

There may be an interval of weeks or months between the invasion of the different serous sacs.

Rheumatism, tubercle, pneumonia, septicæmia and pyæmia are all factors in the ætiology. Subacute and chronic cases are nearly always regarded as tuberculous (Picchini), but there is frequently no direct evidence of the tubercular nature.

The prognosis depends upon the cause. The implication of the pericardium in pneumonia is very unfavourable. Streptococcal and staphylococcal polyorromenitis is often fatal; rheumatic polyorromenitis is much less serious, though complicating the case frequently by adhesions of pleura and pericardium.

Tuberculous peritonitis is fatal in more than 50 per cent., but tuberculous pleurisy in less. The combination is not likely to make the prognosis worse than the more severe inflammation acting alone. But pericarditis ensuing is practically fatal. Picchini's figures are out of 50 cases—22 deaths, 7 improved, 21 cured.

**Treatment.**—Marion[8] urges in the treatment of purulent pleurisy that (1) pus should be evacuated as soon as possible; (2) general anæsthesia should be avoided, on account of syncope and asphyxia consequent thereupon; (3) the point of evacuation should be the most dependent part; (4) the pleura should not be irrigated.

S. H. Perry[9] considers "acute pneumonia" to be "one of the few diseases in which venesection may be used as a curative agent." He considers it of advantage (1) in the early stages of cardiac failure; (2) when complicated by considerable acute bronchitis; (3) in plethoric pneumonia. Among the signs of cardiac failure he includes "a secondary decrease in the loudness of the second sound in the pulmonary area. After the sound has become sharp and accentuated, the tone of it may become dull and muffled, and when this occurs together with increasing dyspnœa, lividity, and commencing pulmonary œdema, it is evidence of the dilatation of the right side of the heart. Venesection is of service when performed before the heart has lost its potential vitality, otherwise it is only palliative. In coincident acute bronchitis venesection relieves the profuse expectoration. In plethoric pneumonia he holds the treatment to be empirical but successful.

Mann[10] has treated tuberculosis with intravenous injections of $\frac{1}{100}$ or $\frac{1}{50}$ gr., increasing to $\frac{1}{3}$ gr. of sod cinnamate or hetol. He claims that the drug induces granulations to spring up round the tuberculous material, absorbing and replacing the necrotic material. Cinnamic Ac. is the efficient constituent of balsam of Peru.

Solis-Cohen[11] testifies to the value of myrtol, obtained by fractional distillation from myrtle oil, in affections of the bronchial mucous membrane. He has seen good results attending its use in bronchorrhœa, dilated bronchi, fibroid tuberculosis with bronchiectatic cavities and in bronchitic asthma. He calls attention to it as a useful adjunct to eucalyptol, terebene, turpentine and sandalwood oil; holding that it should be tried if these, for some reason or other, have failed or seem undesirable: 5 to 15 min. can be given two to five times daily, and should be handled like turpentine. It can be inhaled, or used in powder, tincture or infusion of myrtle leaves.

Braun[11] states that thiocol, a derivative of guiacol (Pot. guiacol sulphonate), contains 52 per cent. of crystallised guiacol, is a white odourless powder with slightly salt and bitter taste, and can be taken without objection for weeks and months. In doses of 5–15 grs. thrice daily appetite improves, expectoration and cough diminish, night sweats cease and sleep becomes sound and restful in phthisical patients. In bronchitis and pulmonary abscess it has been used with excellent results. Besides those mentioned the advantages of this drug over guiacol and creasote are—1. Large quantities can be absorbed by the system without detriment. 2. There is no caustic or irritating effect on the gastric mucous membrane. 3. It can be prescribed in many ways—dry, in aqueous or syrupy solution, in pills or capsules, in cachets, &c.

C. J. Whalen[11] recommends the use of guiacol in pulmonary tuberculosis, giving it internally 5 min. thrice daily in capsules, increasing it by 1 min. daily, and at the same time applying it externally. It is rapidly absorbed through the skin. He also recommends, as a substitute, oil of cloves internally, with the guiacol externally. Inhalation of formaldehyde, 2 to 25 per cent., according to patient's ability to stand it, is also advised. He is disappointed in tuberculin and intrapleural injections of nitrogen.

Weill and Diamantberger[11] recommend the crystallised synthetic guiacol. They administer it hypodermically into the gluteal region, using 15 min., 1 to 8 times daily, of a solution composed thus:—

| | |
|---|---|
| Guiacol (cryst. synthetic) ... ... ... | ʒii. |
| Expressed almond oil (sterilised at 120° C.) | ʒii. |
| Cocaine hydrochlorate ... ... ... | grs. ii. p. |

This is supplemented by means of an enema of milk containing 40-50 min. guiacol oil, painting of the chest, and a pill taken every three or four hours, composed thus:—

| | |
|---|---|
| Guiacol ... ... ... ... | gr. 15 |
| Terpin hydrate ... ... ... | gr. 30 |
| Ac. benzoic ... ... ... | gr. 45 |
| Ext. belladon ... ... ... | gr. 1½ |
| Ext. hyos. ... ... ... | gr. 1½ |

Divide into 100 pills.

The saturation with guiacol is extended over several months, with interruptions of eight to ten days every three weeks. The result is very encouraging in early phthisis, chronic bronchitis with abundant fetid expectoration, in pulmonary gangrene, and in intestinal tuberculosis.

Murrell[12] has had an unfortunate result with cacodylate of sodium, using 1 grain, in pill, thrice daily. Marked symptoms of arsenical poisoning were the result.

Finley[13] publishes notes of a case of pneumo-thorax produced by gas-forming bacteria. He refers to three others, and states that in three of the cases abdominal disease was present (perityphlitis, tubercular peritonitis, and subdiaphragmatic abscess), and one case was due to a wound in the thoracic wall. The organisms found were B. aërogenes capsulatus (2) and B. coli (2). The gas was found to contain much hydrogen.

Fisher[14] reports a case of extensive deposit of phosphate of lime in the lungs. The subject, a woman, aged 32, had consolidation of the right lung, with evidence of local superficial tuberculous disease. In addition the lungs were studded thickly with concentricly laminated nodules of phosphate of lime. So many were the deposits that the lung, where otherwise healthy, sank in water; 98 grs. were removed from one cubic inch of lung tissue. The lungs weighed 57 (right) and 45 ounces. Their composition given by Dr. Munro was

| | |
|---|---|
| Moisture ... ... ... ... | 8 per cent. |
| Albuminoid matter ... ... ... | 7 per cent. |
| Phosphate of lime ... ... ... | 75·7 per cent. |
| Non-nitrogenous organic matter, Carbonates and others ... ... | 9·3 per cent. |

There seemed to be no nucleus to the nodule; they were not in connection with the blood vessels or apparently lymphoid tissue. Dr. Fisher offers the suggestion that they were caused by a "general invasion of the lungs by a microbe of low virulence." There was a superficial resemblance to miliary tuberculosis.

[7] Brit. Med. Journ., Dec. 15th. [8] Med. Rec., Jan. 12th. [9] Birm. Med. Rev., Dec. [10] Med. Rec., Dec. 8th. [11] Merck's Archives, Nov., 1900. [12] Brit. Med. Journ., Dec. 22nd. [13] Dublin Rev., Oct. [14] Lancet, Jan. 26th.

